# Effects of Soaking Tempe in Vinegar on Metabolome and Sensory Profiles

**DOI:** 10.3390/metabo12010030

**Published:** 2022-01-01

**Authors:** Hadi Akbar Dahlan, Yosuke Nambu, Sastia Prama Putri, Eiichiro Fukusaki

**Affiliations:** 1 Department of Biotechnology, Graduate School of Engineering, Osaka University, 2-1 Yamadaoka, Suita 565-0871, Japan; dahlan_hadi_akbar@bio.eng.osaka-u.ac.jp (H.A.D.); yosuke_nambu@bio.eng.osaka-u.ac.jp (Y.N.); fukusaki@bio.eng.osaka-u.ac.jp (E.F.); 2 Industrial Biotechnology Initiative Division, Institute for Open and Transdisciplinary Research Initiatives, Osaka University, Osaka 565-0871, Japan; 3 Osaka University-Shimadzu Omics Innovation Research Laboratories, Osaka University, Osaka 565-0871, Japan

**Keywords:** *tempe*, metabolomics, rate-all-that-apply, triangle test, vinegar

## Abstract

Tempe is a fermented soybean food that is globally renowned for its high protein content. Methods of preparing tempe vary worldwide, and include soaking in vinegar before fermentation. This study aimed to determine the effects of soaking in vinegar by metabolome analysis, gas chromatography/mass spectrometry, and sensory attribute evaluation. Vinegar affected metabolism during tempe fermentation, which led to altered metabolite profiles in the final product. We validated the metabolite profiles of two types of tempe using triangle tests and rate-all-that-apply (RATA) tests, which revealed that the sensory attributes of a golden-brown color, ammonia smell, pleasant smell, salty flavor, and acceptance significantly differed (*p* < 0.05) between the two types of tempe. A high concentration of specific amino acids in the control tempe explained a strong ammonia smell, saltiness, and darker golden-brown sensory attributes. Tempe soaked in vinegar contained high concentrations of metabolites associated with a roasted aroma and cooked meat. In conclusion, most RATA panelists who were being introduced to tempe preferred that soaked in vinegar to the control that was not.

## 1. Introduction

Tempe (tempeh) is a fermented soybean food that has been consumed in Indonesia since the 16th century. It is usually prepared by fermenting soybeans with *Rhizopus* spp. as the starter mold [1]. Tempe is renowned in Indonesia for its high protein, carbohydrate, fat, and dietary fiber contents along with its digestibility and absorption [1]. It is becoming popular among vegans and vegetarians as a meat substitute and is produced in several countries [2,3]. The consumption of tempe in Indonesia accounts for at least 10% of all consumed protein, which is higher than that of chicken eggs (1.25%) or meat (3.15%) [4]. Over 100,000 tempe manufacturers presently range from home to large-scale industry in Indonesia [5], indicating its cultural and economic importance.

Although tempe is nutritious, the product might not be appealing to first-time consumers, especially outside Indonesia, as it has an unpleasant fermented smell. One way to improve tempe acceptance by first-time consumers is to modify how it is processed. While various tempe production methods in Indonesia have been reported [6], the principle has remained unchanged. Tempe is usually made by inoculating dehulled, soaked, and cooked soybeans after incubation with *Rhizopus* spp. for 24 to 48 h. Major variations in production methods are the soaking and cooking steps. Soaking is critical for tempe production because it induces soybean size expansion [7] and promotes acidic fermentation [8,9].

The microbial composition of tempe is determined by ecological factors, such as acidification by lactic acid bacteria during soaking [3]. Therefore, indigenous bacteria play a role in acidification, resulting in a pH decrease during natural soaking. The pH in the natural process ranges from 4.12 to 4.50, which prevents the growth of contaminating bacteria and contributes to low levels of pathogenic and spoilage microorganisms in the final product [4]. On the other hand, acids such as vinegar are applied to lower the pH and control the microbial composition of tempe in industrial processes [10]. Several tempe manufacturers use vinegar [11] although acid is a powerful inhibitor of fungal growth [3] and vinegar might affect the quality of the fermented product. However, little is known about the monitoring and control of tempe production processes and how they affect tempe products.

Metabolomics focuses on variations in metabolite profiles in various types of biological samples [12]. Fermented foods such as coffee have been studied in terms of authentication [13] and geographical origin [14] using metabolomics. A metabolomic study has classified tempe based on geographical origin and different legume materials [2,15]. This technique could be applied to tempe research to determine how adding vinegar during the soaking process affects the metabolomic and sensory profiles of tempe.

This study aimed to define the effects of soaking tempe in vinegar using metabolome analysis, gas chromatography/mass spectrometry (GC/MS), and sensory profiling to provide useful information to the tempe industry. Our results should also provide a viable tempe processing strategy for introducing first-time consumers to tempe.

## 2. Results and Discussion

### 2.1. Metabolite Profiles of Tempe with and without Soaking in Vinegar over Time

**Figure 1:**
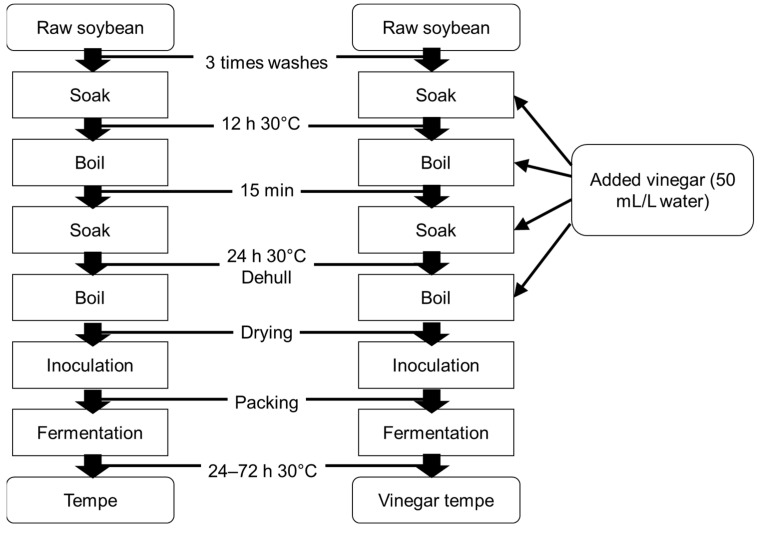
Workflow of production of control and vinegar-treated tempe using Rumah Tempe Indonesia (RTI) method.

The amount of time required to completely ferment tempe soaked without (control) and with added vinegar differed (Figure 1); the desired texture of control and vinegar-treated samples was achieved in 48 and 72 h, respectively (Figure S1). Fermentation was delayed because vinegar inhibits fungal growth [4]. Since the metabolites of tempe change according to pretreatment and duration of fermentation [16], samples of tempe soaked with and without vinegar collected at 24 and 48 h, and at 24, 48, and 72 h were analyzed by GC/MS. We detected 115 compounds in the tempe samples that were annotated, including 32 that were unknown (Table S1). The processed GC/MS dataset was assessed using multivariate analysis, and differences between the samples were determined by principal component analysis (PCA).

**Figure 2:**
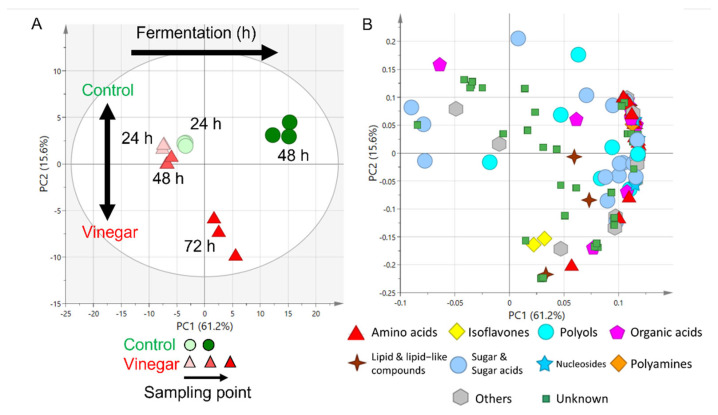
Principal component analysis (PCA) of tempe fermented without and with vinegar for 24 and 48 h, and for 24, 48, and 72 h, respectively. (**A**) Score plot. Circles and triangles of different colors represent tempe soaked without (control) and with vinegar, sampled at various points (**B**) Loading plot. Different shapes and colors represent metabolite classes.

The results of the PCA revealed variations of 61.2% and 15.6% for PC1 and PC2, respectively (Figure 2A). Based on how the samples were grouped, the axis of PC1 shows the progression of fermentation time, and the axis of PC2 shows differences between soaking the tempe with and without (control) vinegar as fermentation progressed. The metabolite profiles differed between control tempe soaked for 48 h without vinegar (control) and that soaked with vinegar for 72 h.

Figure 2B shows the PCA loading plots. Most metabolites, including those of amino acids, isoflavones, polyols, sugar and sugar acids, nucleosides, polyamines, and organic acids, were positioned on the positive side of PC1. The class of metabolites detected coincided with previous tempe study [17]. These results indicated that the amount of these metabolites increased as mycelia formed throughout tempe fermentation, regardless of the soaking conditions. This coincided with previous reports of increased isoflavones [18] and amino acids [19] during tempe fermentation. The metabolites of arabitol, n-propylamine, glucono-1,5-lactone, malonic acid, sorbose, gluconic acid, fructose, psicose, and tagatose were located on the negative side of PC1, indicating decreasing quantities as tempe fermented. Most of these metabolites were derived from sugars and sugar-alcohols. The decrease in sugar-based metabolites is reasonable because they are degraded during the formation of *Rhizopus* sp. mycelia [20]. Table S2 shows the loading values of all detected and annotated metabolites. Tempe soaked without and with vinegar was fermented for 48 and 72 h, respectively, in subsequent sensory analysis, based on the increase of most metabolites.

### 2.2. Sensory Differences between Tempe Fermented with and without Vinegar Assessed Using Triangle Tests

We investigated sensory differences between tempe fermented with and without vinegar using triangle tests (Table 1). The objective of the sensory triangle test is to determine the existence of perceptible sensory differences between two products [21]. The results showed that 15 of 25 panelists could discriminate tempe fermented with and without vinegar. The alpha (α) risk table of statistics showed that the sensory difference between tempe fermented with and without vinegar was detectable at an α-risk level of 0.01 [22]. 

The triangle test result was notable because the panelists included individuals who did not regularly consume tempe, and they were able detected a perceptible sensory difference between the two types of fermented tempe. However, the triangle test does not describe sensory attributes that differ between two samples. Therefore, we investigated differences in sensory attributes between tempe soaked with and without vinegar using rate-all-that-apply (RATA) tests.

### 2.3. Sensory Attributes of Tempe Fermented with and without Vinegar Using Rate-All-That-Apply (RATA) Tests

Table S3 shows the 16 sensory attributes included in the RATA test. These attributes are based on the categories of color (golden-brown), texture (compact and crispy), aroma (beany, tempe-specific, alcohol, pleasant, yeast, and ammonia), taste (sweet, sour, salty, bitter, umami, astringent), and hedonic (acceptance).

**Figure 3:**
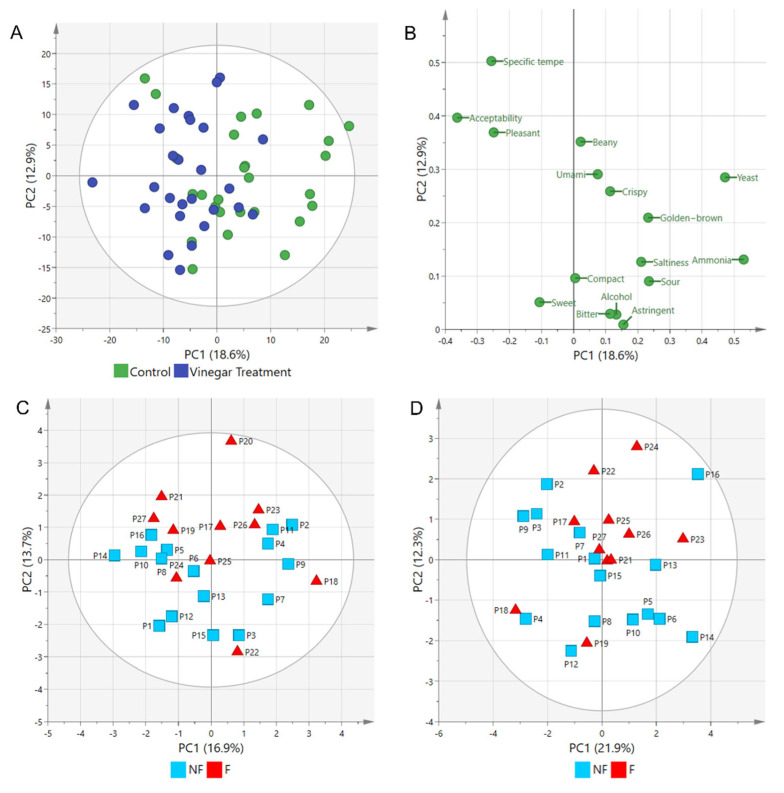
Principal component analysis (PCA) of rate-all-that-apply (RATA) tests of two types of tempe and panelist performance. (**A**) Score plot. (**B**) Loading plot. Panelist performance was based on familiarity with tempe sensory characteristics before RATA tests. Score plots of panelist performance for tempe fermented without (control) (**C**) and with (**D**) vinegar.

Among the 27 panelists who participated in the RATA tests, 11 were familiar with tempe sensory characteristics, and 16 were not. Table S4 and Table 2 show the characteristics of the 27 panelists and the descriptive statistics of the sensory attributes in the test. Based on descriptive statistics (Table 2), all sensory attributes significantly differed (*p* < 0.05, Shapiro–Wilk tests) and were transformed to create a PCA plot, which shows variations of 18.6% and 12.9% in PC1 and PC2, respectively (Figure 3A). The small variation (%) in both principal component axes indicate small perceptible sensory differences based on the 16 attributes. However, the clusters of tempe soaked with and without vinegar on the negative and positive sides, respectively, of PC1 showed that most panelists could differentiate the sensory attributes of tempe soaked without and with vinegar.

Figure 3B shows the PCA loading plot of the RATA test. The positions of the 16 sensory attributes indicated that most of them were placed higher in control tempe. Only tempe-specific smell, pleasant smell, sweet taste, and acceptability were higher for tempe fermented with vinegar. Another notable result from the RATA test was the grouping of the panelists based on their familiarity with tempe sensory characteristics. Figure 3C,D shows PCA plots of the RATA panelists for the two types of tempe. Most of the panelists who were familiar with the sensory characteristics of tempe were grouped together on the positive side of PC2. This trend was similar for tempe soaked without (Figure 3C) and with (Figure 3D) vinegar. The group differences between the panelists can explain the non-normal distribution of the RATA results. The results also indicated that preferences differed between panelists who were introduced to tempe during the present study, and those who were already familiar with its sensory characteristics.

**Figure 4:**
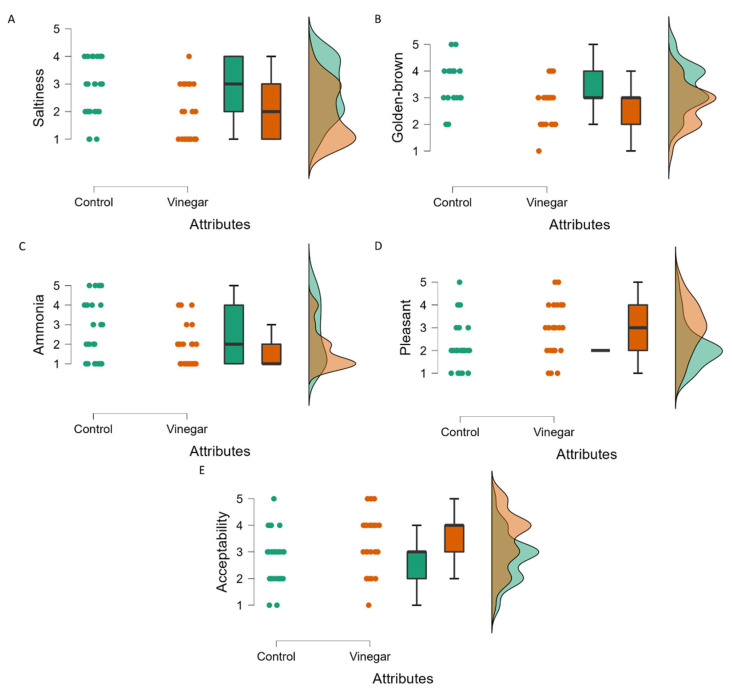
Raincloud plots for five significantly different sensory attributes (*p* < 0.05). (**A**) Saltiness, (**B**) golden-brown color, (**C**) ammonia smell, (**D**) pleasantness and (**E**) acceptability. Raincloud plots comprise a combination of dot, box and distribution plots.

The statistical analysis of the 16 sensory attributes showed that only golden-brown color, saltiness, ammonia smell, pleasant smell, and acceptability attributes (Table 2) achieved statistical significance (*p* < 0.05). All five significant sensory attributes were analyzed using raincloud plots (Figure 4), as these can visualize RATA panelist choices in terms of raw data, box plots, and probability density [23]. Three of the sensory attributes, namely saltiness (Figure 4A), golden-brown (Figure 4B), and ammonia (Figure 4C), were significantly higher (*p* < 0.05) in the control tempe (Table 2). This is evident in box and dot plots, where more panelists scored these three significant sensory characteristics higher in the control tempe. The panelists were questioned about their preferences for colors in a range from light to darker golden-brown, whether they associated the taste of tempe with sodium chloride (salty), and whether the tempe provoked a sharp sensation of nasal mucous associated with ammonia. The results of the dot and distribution plots showed that most panelists rated the control tempe as salty, golden-brown, and smelling like ammonia.

The non-normal distribution of the panelist results was due to the 11 and 16 panelists who were respectively familiar and unfamiliar with tempe sensory attributes, and judged the attributes differently. As most panelists were judging tempe sensory characteristics for the first time in the present study, we deduced that they found the tempe fermented with vinegar less salty, lighter in color, and smelled less of ammonia than the control, whereas those familiar with tempe sensory characteristics had different opinions.

In addition to these three significant sensory characteristics, pleasant smell and hedonic acceptance attributes were also significant. Panelists selected which types of tempe were associated with good connotations and a pleasant smell attribute. The hedonic acceptance attribute describes acceptance of tempe as an overall value, concurrent with all attributes. The pleasant smell (Figure 4D) and hedonic acceptance (Figure 4E) attributes were significantly higher (*p* < 0.05) in the tempe fermented with vinegar (Table 2). A boxplot for pleasant smell in control tempe was not created due to similar values for the 25th and 75th quartiles of the data. However, the dot and distribution plots were sufficient to visualize differences in panelist choice between the two types of tempe. The dot and skewed distribution plot show that most panelists felt that the smell was more pleasant for tempe soaked in vinegar than the control (Figure 4D). This result coincides with the ammonia smell (Figure 4C), as most panelists felt that tempe fermented with vinegar smelled less of ammonia.

The raincloud plots (Figure 4E) show that most panelists preferred the tempe soaked with rather than without vinegar, which was reasonable because the former had less of a salty taste and ammonia smell, and a more pleasant smell. The results also revealed that the panelists who were new to tempe preferred lighter over darker, golden-brown tempe. These findings indicated that soaking tempe in 50 mL/L of vinegar during fermentation is a viable processing strategy for introducing tempe to people without knowledge of its sensory characteristics. Figure S2 shows raincloud plots for all other sensory attributes.

### 2.4. Metabolite Profile of Control Tempe and Vinegar-Treated Tempe

**Figure 5:**
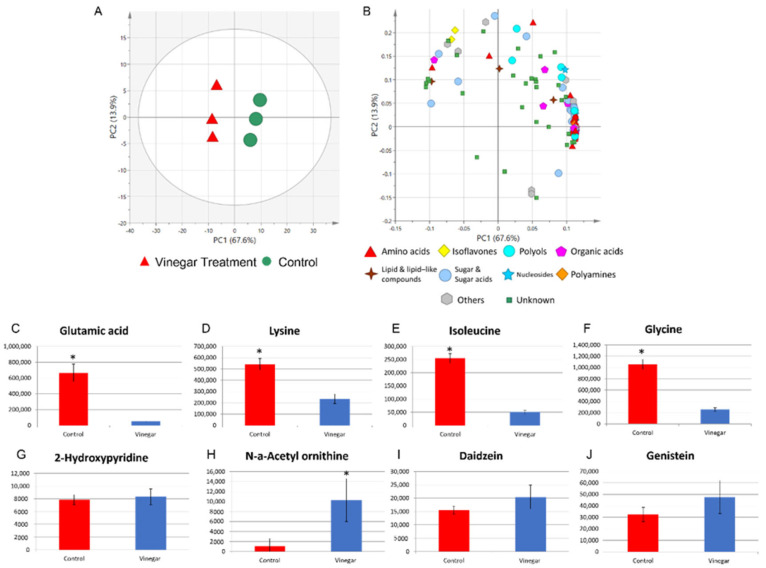
Principal component analysis of tempe soaked without and with vinegar and bar graph of selected metabolites. (**A**) Score and (**B**) loading plots of tempe soaked without (control) and with vinegar. Bar graph shows relative intensity of (**C**) glutamic acid, (**D**) lysine, (**E**) isoleucine, (**F**) glycine, (**G**) 2-hydroxypyridine, (**H**) N-*a*-acetylornithine, (**I**) daidzein, and (**J**) genistein. * *p* < 0.05 (significantly different, Student’s *t*-test).

The different intensity of sensory attributes among the RATA panelists regardless of tempe familiarity is due to the metabolite profile of the control and tempe soaked in vinegar. The results of the PCA of tempe soaked without and with vinegar before fermentation showed variations of 67.6% and 13.9% for PC1 and PC2, respectively (Figure 5A). This large variance and clear differences in group position were used to visualize differences between the metabolomic profiles of tempe soaked with and without vinegar.

The loading plot shows that both types of tempe contain different amounts of various metabolite classes (Figure 5B). Most metabolites were positioned on the positive side of PC1, indicating that the control tempe contained more amino acid metabolites, polyols, organic acids, nucleosides, sugars, and other classes compared to the tempe soaked in vinegar (Figure S3). Polyol and amino acids accumulate during tempe fermentation [1,24]. In contrast, the metabolites on the negative side of PC1 represent tempe soaked in vinegar, and comprised the degradation products of raffinose, glutamine, 2-hydroxypyridine, oxalate, fructose-6-phosphate, genistein, daidzein, phosphate, sucrose, myristic acid, ornithine, N-a-acetyl ornithine, and sorbose. Raffinose and sucrose are commonly degraded during tempe fermentation [20]. However, our results showed that soaking in vinegar decreased the degradation rate of various sugar metabolites. Similarly, the retention of daidzein and genistein in soybeans is affected by the type of water in which the tempe is soaked [25]. Our results showed that although tempe soaked in vinegar had fewer amino acid metabolites, it had more daidzein and genistein. Table S4 and Figure S3 show the loading values of all metabolites and a bar graph of all annotated metabolites, respectively.

The different metabolite profiles can explain the sensory differences between the two types of tempe; the amino acid metabolite class differed the most between the two groups (Figure 5B). One example is glutamic acid (*p* < 0.05) (Figure 5C), which is partly responsible for the umami taste [26] and saltiness in other fermented foods [27]. However, the RATA panelists found no significant differences in umami sensory attributes between the two types of tempe (Figure S2), whereas the high relative intensity of glutamic acid coincided with the RATA intensity of high saltiness in the control tempe. Control tempe contained more glutamic acid, lysine, isoleucine, and glycine (Figure 5D–F). These three amino acids are highly reactive and contribute to the Maillard reaction [28]. The high relative intensity of free amino acid reactions with reducing sugars in the control tempe would result in a deeper golden-brown color [29] as perceived by the panelists.

In addition to the high contents of amino acid metabolites in control tempe, the dominant metabolites can also be associated with sensory characteristics in tempe soaked in vinegar. The metabolite 2-hydroxypyridine (Figure 5G) elicits a roasted smell [30]. Similarly, N-a-acetyl ornithine (Figure 5H), which was significantly more abundant (*p* < 0.05) in the tempe soaked in vinegar, is associated with the taste of cooked meat [31]. Although the amount of 2-hydroxypyridine was not significantly higher in tempe soaked in vinegar than in control tempe, the combination with significantly higher content of N-a-acetyl ornithine might have influenced the RATA panelists to rate the hedonic acceptability sensory attributes vinegar-treated tempe more highly. The isoflavones daidzein (Figure 5I) and genistein (Figure 5J) can also elicit a bitter taste [32]. However, our RATA panelist results found that the bitter taste was comparable between the two types of tempe (Figure S2). The contents of daidzein and genistein were not significantly higher in tempe soaked in vinegar compared with the control. However, the RATA results showed that vinegar can elicit a similar level of bitter taste even though the intensity of bitter-tasting amino acids such as isoleucine are lower in control tempe [33]. Considering all the significant sensory attributes, the decreased ammonia smell and the salty taste combined with a pleasant smell and a lighter golden-brown color led most panelists to accept the tempe soaked in vinegar more easily than the control.

In summary, metabolite profiling using GC/MS showed that vinegar altered metabolite production during tempe fermentation, which led to different metabolite profiles in the final products. The different metabolite profiles of tempe soaked with and without vinegar were validated using triangle tests, which did not reveal sensory differences, and RATA tests, which complemented the triangle tests by specifying distinctive sensory attributes between the two types of tempe. The RATA test results showed that preferences differed between panelists who were familiar and unfamiliar with the sensory characteristics of tempe. Among the 16 sensory attributes tested, golden-brown color, ammonia smell, pleasant smell, salty taste, and acceptance significantly differed (*p* < 0.05) between the two types of tempe. The non-normal distribution of the RATA results indicated that most panelists who were new to the sensory characteristics of tempe preferred tempe soaked in vinegar over control tempe. The metabolomic findings of the two types of tempe indicated that high concentrations of specific amino acid metabolites in control tempe were responsible for the ammonia smell, salty taste, and golden-brown color of control tempe. In contrast, tempe soaked in vinegar contained more metabolites associated with the taste of roasted and cooked meat. The panelists indicated that tempe soaked in vinegar smelled more pleasant than control tempe, probably because of the differences in metabolite profiles. Thus, the panelist rates of acceptance of sensory attributes were higher for the tempe soaked in vinegar. We found that soaking in vinegar could be a feasible strategy for introducing tempe to new consumers with no prior knowledge of its sensory characteristics. To the best of our knowledge, this is the first study to reveal changes in the metabolite profiles and sensory attributes of tempe soaked in vinegar before fermentation.

## 3. Materials and Methods

### 3.1. Sample Preparation

A tempe–mold starter culture was obtained from Raprima brand (Aneka Fermentasi Industri, Bandung, Indonesia). The raw materials were Japanese soybeans of the Yukihomare variety (grown in Ishikari, Japan). Tempe was washed, soaked, and boiled in mineral water (Suntory, Tokyo, Japan), and vinegar was purchased from Mizkan Ltd. (Handa, Japan).

Tempe was processed according to the Rumah Tempe Indonesia (RTI) method (Figure 1) [8], in which tempe is soaked and boiled in vinegar (50 mL/L of mineral water). The tempe was packed, then fermented in an incubator (EYELA, Tokyo, Japan) at 30 °C for 24 to 72 h until the desired texture was achieved. Metabolite changes during fermentation were determined by sampling tempe with and without vinegar processing at 24 and 48 h, and 24, 48, and 72 h, respectively.

### 3.2. Metabolite Extraction and Derivatization

Frozen tempe was milled into a fine powder, then 15 mg of lyophilized tempe was vortex-mixed with 1 mL of a 5:2:2 (v/v/v) ratio of methanol, ultrapure water (both from Wako Pure Chemical Industries, Osaka, Japan) and chloroform (Kishida Chemical Co. Ltd., Osaka, Japan) and the internal standard ribitol (100 µg/mL). The mixture was separated by centrifugation at 4 °C and 11,740× *g* for 3 min, then 400 µL of supernatant was vortex-mixed with ultra-pure water (400 µL) in 1.5-mL microtubes to increase separation between the polar and non-polar phases. The mixture was centrifuged for 3 min at 4 °C, 11,740× *g* for 3 min. The aqueous phase (400 µL) was transferred to fresh 1.5 mL microtubes with pierced caps. Pooled quality controls (QCs) were prepared by mixing 200 µL of the aqueous phase from all samples. The solvent was evaporated using a Spin Dryer Standard (Taitec Co., Kyoto, Japan) for 2 h at room temperature. The evaporated samples were lyophilized overnight (Taitec Co.). Methoxyamine hydrochloride (100 µL, 20 mg/mL in pyridine) was added on the following day and incubated at 30 °C and 161× *g* for 90 min. Subsequently, 50 µL N-methyl-N-(trimethylsilyl)-trifluoroacetamide (MSTFA) (GL Sciences Inc., Tokyo, Japan) was added and the mixture was incubated at 37 °C and 161× *g* for 30 min before GC-MS analysis.

### 3.3. GC/MS Analysis

Samples were derivatized before GC/MS to analyze non-volatile metabolites. Lyophilized samples were oximized with 100 µL of methoxyamine hydrochloride (20 mg/mL in pyridine) (Sigma-Aldrich, St. Louis, MO, USA) then shaken at 161× *g* in an incubator at 30 °C for 90 min. The samples were silylated with 50 µL of N-methyl-N-trimethylsilyl-trifluoroacetamide (MSTFA) (GL Sciences Inc.), then incubated at 37 °C for 30 min at 161× *g*.

Metabolites were profiled using a GC/MS-QP2010 (Shimadzu, Kyoto, Japan) equipped with a 0.25 × 30 mm, 0.25-µm Inert Cap 5 MS/NP column (GL Sciences Inc., Tokyo, Japan), and an AOC-20i/s autosampler (Shimadzu, Kyoto, Japan) under the conditions applied in a previous study of coffee metabolomics [13]. The reference for retention time comprised a standard alkane mixture (C8–C40) that was injected before sample analysis.

### 3.4. Metabolome Data Analysis

The GC/MS data were converted into ANDI files (*cdf) using GCMS Solution software (Shimadzu, Kyoto, Japan), then peaks were detected, the baseline was corrected, and peak retention times were aligned using MS-DIAL v. 3.70 (Riken, Kanagawa, Japan) [34]. Peaks were annotated based on the retention index and MS information in the GL-Science Database. The GL-Science Database is a public metabolite library shared by RIKEN (http://prime.psc.riken.jp/compms/msdial/main.html, accessed on 23 December 2021). Data patterns were searched based on differences and similarities by multivariate analysis using SIMCA-P+ v.13 (Umetrics, Umeå, Sweden) for PCA without data transformation. The relative intensity of each metabolite was normalized by comparison with that of the internal standard as an explanatory variable. The data were auto-scaled to reduce the mask effect of abundant metabolites. The class for each annotated metabolite was determined using the Human Metabolome Database (https://hmdb.ca/, accessed on 23 December 2021).

### 3.5. Cooking and Serving Method for Triangle and Rate-All-That-Apply (RATA) Tests

We prepared tempe as described in sub-Section 2.4. Tempe soaked with and without vinegar was sampled 48 and 72 h, respectively, cut into cubes, cooked in an air fryer (Innsky, Shenzhen, China) for 5 min at 200 °C and served in plastic cups labeled with random three-digit codes.

### 3.6. Triangle Test

A panel of 25 assessors (average age, 26 y; female, *n* = 12; male, *n* = 13 men; Japanese, *n* = 17; non-Japanese *n* = 8) were trained on the five-basic taste test. Three tempe samples were simultaneously presented in the triangle test. Two were the same type, and one was a different type [21]. The test was applied twice with two sets of test samples per test, comprising samples A (control) and B (soaked in vinegar). The combinations and orders were randomized in each set for each panelist. Panelists recorded sample codes for which they identified an overall sensory difference. Only panelists who identified differences between the two types of tempe in both sets of tests were considered correct. Figure S4 shows the test questionnaire. The results of the triangle tests were analyzed using α risk statistics based on the British Standard ISO 4120-2004 [21,22].

### 3.7. Rate-All-That-Apply (RATA) Tests

A panel of 27 assessors (average age, 29.5 y; female, *n* = 10, male, *n* = 17; Japanese, *n* = 19; non-Japanese, *n* = 8) participated in these tests. During selection, the panelists were questioned about their familiarity with the sensory characteristics of tempe. Regardless of their responses, the panelists were familiarized for 30 and 60 min with the 16 sensory attributes of tempe. Tempe samples that were soaked without (control; A) and with (B) vinegar were presented along with a questionnaire with a list of 16 sensory attributes. The combinations and order of the samples were randomized (AB and BA) for each panelist, and they rated the intensity of each attribute using a 5-point scale. Figure S5 shows the test questionnaire, and Table S3 describes each of the 16 attributes that were developed based on previous work [35,36,37,38,39]. The intensity of the RATA data were described, and were non-normally distributed. The data were analyzed using Mann–Whitney U-tests for statistical comparisons [40], and visualized using raincloud plots [23] and principal component analysis. Data were center-scaled, power transformed, then assessed by PCA using SIMCA-P+ version 13 (Umetrics). Raincloud plots were created using JASP software v. 0.15 [41].

## Figures and Tables

**Table 1:** Details and results of triangle test.

Number of panelists	25 (13 male, 12 female)
Sample	2 × Control tempe (48 h) 1 × Vinegar-treated tempe (72 h)
Number of panelists successful distinguishing vinegar-treated tempe	15
Alpha risk study (Differentiation test)	Significantly different (α = 0.01)

**Table 2:** Descriptive statistics of sensory attributes in RATA tests.

Sensory Attributes	Tempe Types	Median	Mean	Std. Deviation	*p*-Value of Shapiro–Wilk	*p*-Value of Mann–Whitney U
Golden-brown	Control	3	3.37	0.839	0.004	0.009
	Vinegar	3	2.741	0.764	0.001	
Compact	Control	3	3.148	0.907	<0.001	0.503
	Vinegar	3	3.296	0.869	0.003	
Crispy	Control	3	2.481	0.975	0.006	0.207
	Vinegar	2	2.148	0.864	0.002	
Beany	Control	3	3.037	1.16	0.032	0.666
	Vinegar	3	3.148	0.949	0.009	
Specific tempe	Control	3	3.222	1.086	0.027	0.359
	Vinegar	4	3.481	0.975	0.006	
Alcohol	Control	2	1.815	0.921	<0.001	0.316
	Vinegar	1	1.556	0.751	<0.001	
Pleasant	Control	2	2.148	0.989	<0.001	0.003
	Vinegar	3	3.000	1.109	0.036	
Yeast	Control	3	2.963	1.315	0.024	0.228
	Vinegar	3	2.519	1.189	0.012	
Ammonia	Control	2	2.593	1.526	<0.001	0.041
	Vinegar	1	1.741	1.023	<0.001	
Sweet	Control	2	1.852	0.77	<0.001	0.227
	Vinegar	2	2.333	1.271	0.002	
Sour	Control	2	2.185	1.145	<0.001	0.063
	Vinegar	1	1.593	0.694	<0.001	
Saltiness	Control	3	2.741	1.023	0.002	0.006
	Vinegar	2	1.926	0.958	<0.001	
Bitter	Control	2	2.296	1.031	0.004	0.392
	Vinegar	2	2.074	1.072	<0.001	
Umami	Control	3	2.963	0.98	0.024	0.453
	Vinegar	3	2.704	1.068	0.002	
Astringent	Control	2	2.259	1.13	0.001	0.231
	Vinegar	2	1.926	1.072	<0.001	
Acceptability	Control	3	2.778	0.934	0.017	0.026
	Vinegar	4	3.407	1.083	0.02	

SD, standard deviation. Mann–Whitney U tests.

## Data Availability

The data presented in this study are available in the supplementary materials.

## Supplementary Materials

The following are available online at https://www.mdpi.com/article/10.3390/metabo12010030/s1. Figure S1. Progression of fungal growth during fermentation. Control sample fermented for (**A**) 24 and (**B**) 48 h. Sample soaked in vinegar for 24 (**C**), 48 (**D**), and 72 (**E**) h. Figure S2. Raincloud plots of 16 sensory attributes. Figure S3. Bar graph of all annotated metabolites in tempe soaked without (control) and with vinegar. Figure S4. Triangle test questionnaire. Figure S5. RATA test questionnaire. (**A**) Instructions for RATA test. (**B**) Answer sheet. Table S1. Annotated peaks in all tempe samples by GC/MS analysis. Table S2. Loading values of all detected and annotated metabolites in all tempe samples. Table S3. List of 16 sensory attributes. Table S4. Profile of RATA panel.
