# Supplementary figures and images for: Effects of Soaking Tempe in Vinegar on Metabolome and Sensory Profiles
Source: Metabolites. 2022 Jan 1;12(1):30. doi: 10.3390/metabo12010030 (PMC8781261; doi:10.3390/metabo12010030)

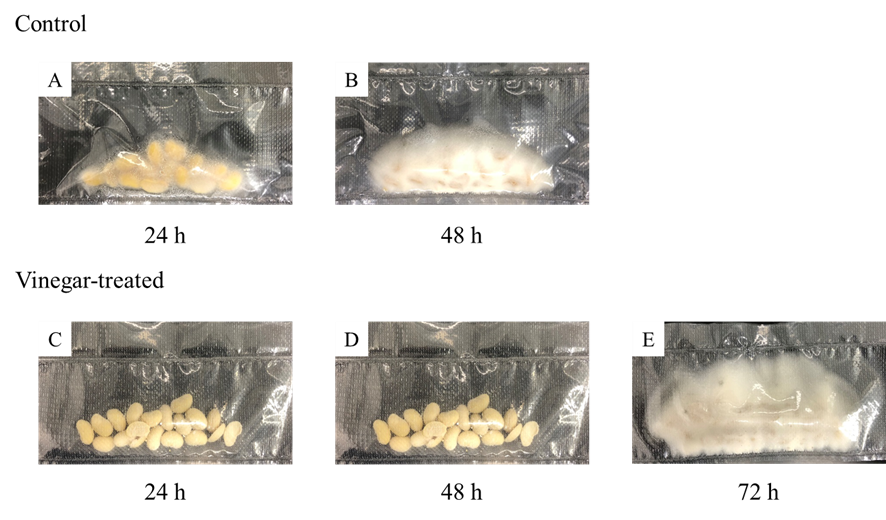

Supplement: Supplementary file 1 [file metabolites-12-00030-s001.zip › Supplementary Fig Metabolites/Fig S1.tif]

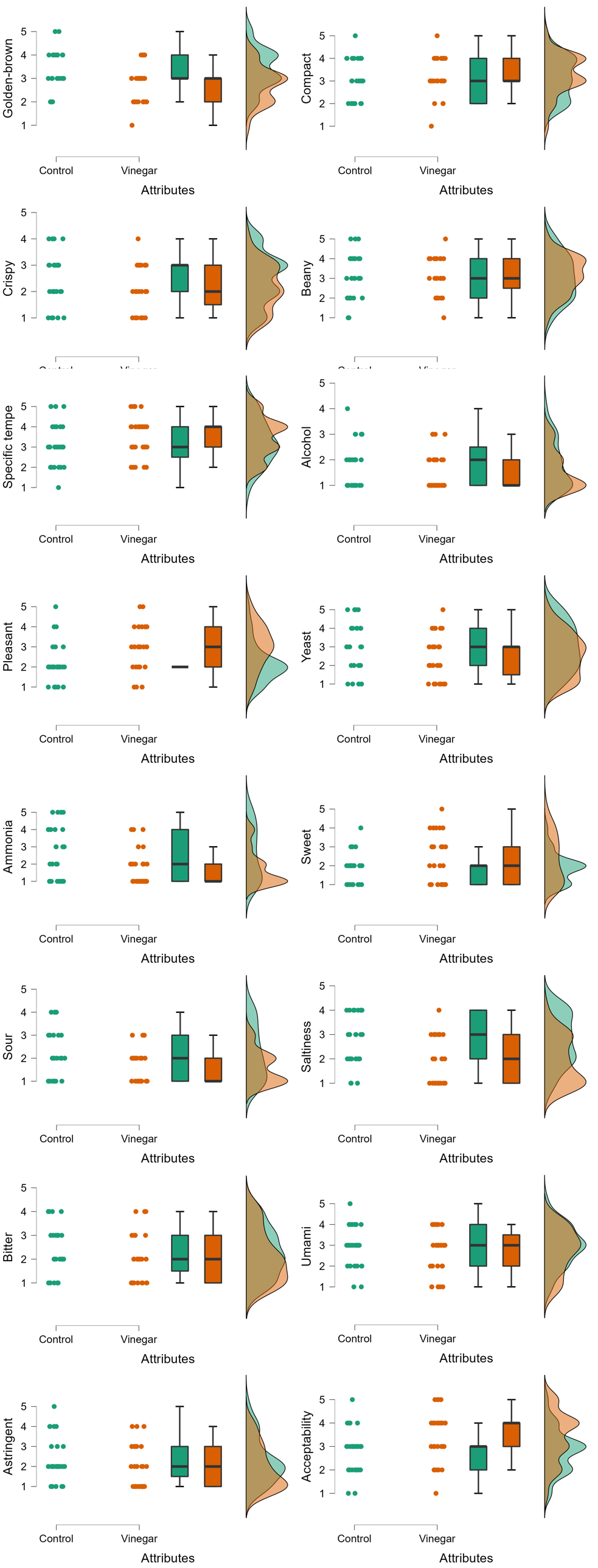

Supplement: Supplementary file 1 [file metabolites-12-00030-s001.zip › Supplementary Fig Metabolites/Fig S2.tif]

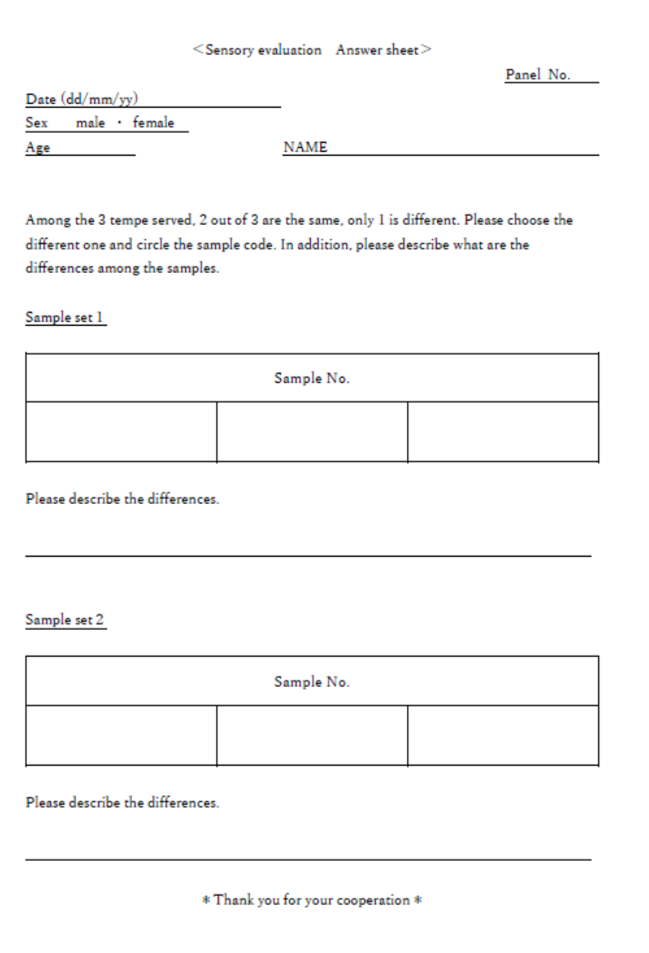

Supplement: Supplementary file 1 [file metabolites-12-00030-s001.zip › Supplementary Fig Metabolites/Fig S4.tif]

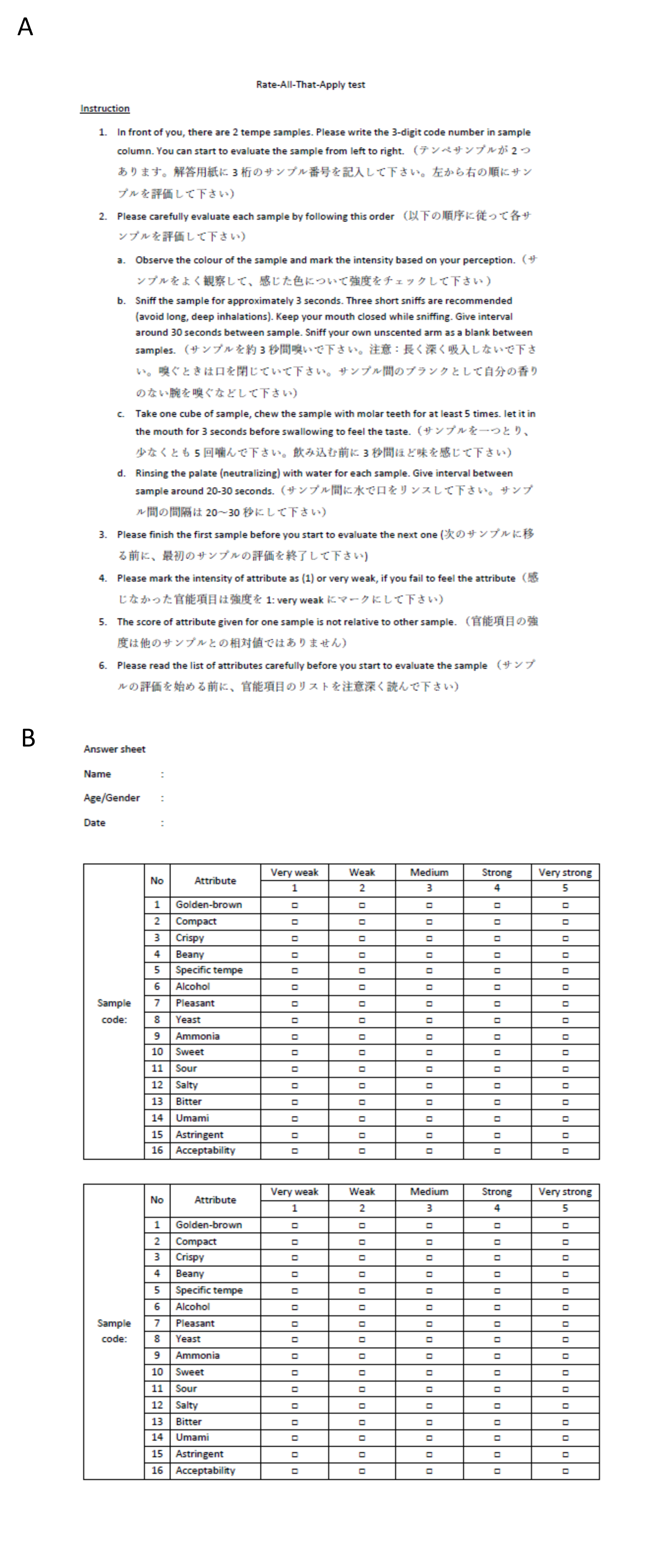

Supplement: Supplementary file 1 [file metabolites-12-00030-s001.zip › Supplementary Fig Metabolites/Fig S5.tif]
